# First description of *Trypanosoma cruzi* human infection in Esmeraldas province, Ecuador

**DOI:** 10.1186/1756-3305-7-358

**Published:** 2014-08-06

**Authors:** Ángel Guevara, Juan Moreira, Hipatia Criollo, Sandra Vivero, Marcia Racines, Varsovia Cevallos, Rosanna Prandi, Cynthia Caicedo, Francisco Robinzon, Mariella Anselmi

**Affiliations:** Laboratorio de Parasitologia Molecular y Medicina Tropical, Centro de Biomedicina, Carrera de Medicina, Universidad Central del Ecuador, Sodiro N14121 e Iquique, Quito, Ecuador; CECOMET, Nelson Estupiñan Bass 210 y Luis Tello, Esmeraldas, Ecuador; Instituto Nacional de Investigación en Salud Pública, Entomología, Ministerio de Salud del Ecuador, Sodiro y Yaguachi, Quito, Ecuador; Centro de Enfermedades Tropicales, Hospital Sacro Cuore, Verona, Negrar Italia

**Keywords:** *Triatoma dispar L*, *Trypanosoma cruzi*, Ecuador, Esmeraldas, Awá population, Chagas disease

## Abstract

Chagas disease was described in Ecuador in 1930 in the province of Guayas and thereafter in various provinces. Triatomine were reported in the province of Esmeraldas but no human infection has been described. Here we report the first evidence that the disease does exist in the province of Esmeraldas. In indigenous Awá communities located in the northwest jungle of the Esmeraldas province, 144 individuals were tested using ELISA and PCR for *T.cruzi* of which 5 (3.47%) were positive. Twenty eight triatomine were collected, 27 were *Triatoma dispar* and 1 *Pastrongylus rufotuberculatus*, *T.cruzi* was detected in 11 (42.3%) of 26 insects.

## Letter

Chagas disease or American trypanosomiasis, is caused by an infection by the protozoan hemoflagellate *Trypanosoma cruzi*, which is transmitted to humans through bites of infected triatomine insects. The infection is widespread throughout Latin America although an increasing number of cases in non-endemic countries have been described [1]. In Ecuador, human *T.cruzi* infections have been observed in different provinces since 1930 [2, 3]. However, in the Esmeraldas province, although triatomine insects were reported, autochthonous cases of *T.cruzi* human infection have not been documented [2]. The province of Esmeraldas, bordering the southern regions of Colombia, is located in the northwest of the country. Most of the inhabitants are of African descendant with dispersed indigenous populations such as the Epera, Chachi and Awá. The latter are also called *kwaike*r, speak their own language (*Awá pit*) and live in remote areas, isolated from any urban areas. Reports from individuals from three Awá’s villages: Mataje Alto (17 N 0772280, UTM 0134144, 221 m), Pambilar (17 N 0766542, UTM 0124494, 144 m) and Balsareño (17 N 0761275, UTM 0128009, 44 m) revealed the presence of triatomine insects in their homes and in peridomestic areas. Therefore, a protocol to study insects and human blood of Awá population was prepared and approved by the Bioethics Committee COBI-ASFORUM (Federalwide Assurance FWZ00002482, IEC IORG0001932, IRB00002438, IEPIDNPI 125754-12/132854-13) in Quito-Ecuador.

**Figure 1:**
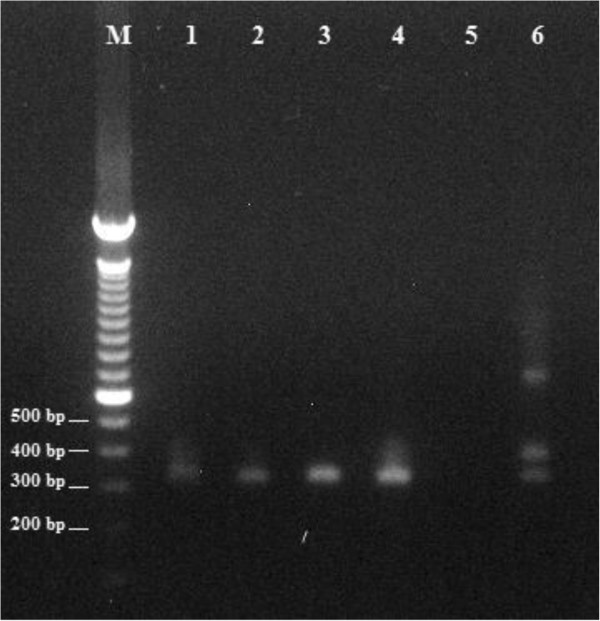
**Agarose gel showing PCR products 330 bp amplified with** ***T.cruzi*** **specific primers Tc121 y Tc122.** M: DNA molecular markers. Lane 1 – 4 samples from *Triatoma dispar* L. Lane 5 negative control. Lane 6 positive control.

We collected twenty-eight insects and sent them to the laboratory for identification and detection of *T.cruzi* infection. Twenty-seven of the insects were *Triatoma dispar* Lent and one was *Pastrongylus rufotuberculatus* according to conventional taxonomic keys. Since the insects were not optimally preserved, it was impossible to detect living forms of trypanosomes using microscopic techniques. Specific PCR for *T.cruzi* DNA was performed according to previously described procedures [4, 5] in 26 of 28 specimens collected and 11 (42.3%) were positive (Table 1, Figure 1).

**Table 1:** **Triatomine insects collected in Awá’s communities of northwest jungle of Esmeraldas province, Ecuador**

Community	No	Species	Habitat
Pambilar	17	*Triatoma dispar*	Peridomestic
Balsareño	10	*Triatoma dispar*	Domestic
Balsareño	1	*Pastrongylus rufotuberculatus*	Domestic

**Figure 2:**
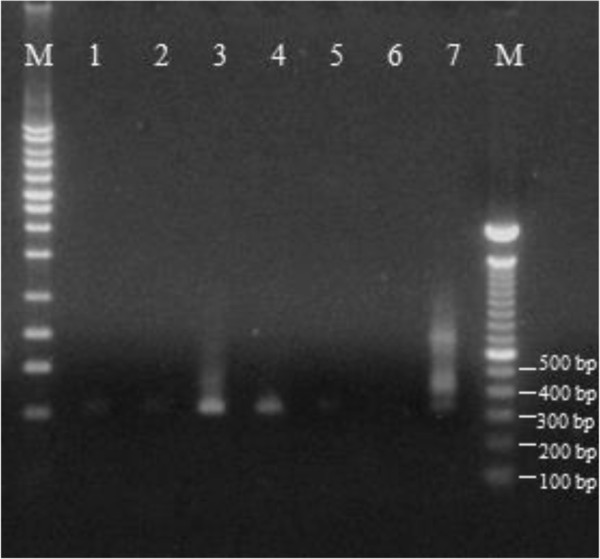
**Agarose gel showing PCR products 330 bp amplified with** ***T.cruzi*** **specific primers Tc121 y Tc122.** M: DNA molecular markers. Lane 1 – 5 blood samples from Awá infected people. Lane 6 negative control. Lane 7 positive control.

In two Awá’s villages (Pambilar and Balsareño), 144 blood samples were obtained with informed consent and sera were analyzed for antibodies against *T.cruzi* utilizing three different serological tests according to manufacturer’s instructions. The three different commercial ELISA tests showed high absorbance values indicating a strong anti-*T.cruzi* IgG response in 5 (3.47%) of 144 samples tested. *T.cruzi* specific PCR [6, 7] was also positive in all 5 *T.cruzi* ELISA positive individuals (Table 2, Figure 2).

**Table 2:** **Results expressed in absorbance values from human sera samples with three different ELISA tests**

Sample code	Age	Gender	Biokit (ELISA)®	Bioschile (ELISA)®	Chagatest (ELISA)®
Pamb 10	21	Male	1.82	1.50	0.62
Pamb 12	29	Female	> 2.0	> 2.0	2.0
Pamb 18	48	Female	> 2.0	> 2.0	> 2.0
Pamb 59	62	Male	1.84	1.96	2.0
Bals 37	32	Male	> 2.0	> 2.0	> 2.0

Cut-off value: Biokit negative <0.9; Bioschile negative < 0.9; Chagatest negative < 0.230.

The present study demonstrates human *T.cruzi* infection in the northwest jungle of Ecuador. The extent of the infection as well as the associated Chagas disease pathology, if any, in the Awá population remains to be determined. In any case, our findings should alert Ecuadorian health authorities to start an integrated strategy to provide treatment and prevention measures to avoid further transmission. *Triatoma dispar* Lent is considered as a sylvatic species and has been reported in the northeast jungle of Ecuador [8] but not in the northwest jungle, in this letter 27 (96%) of 28 triatomine insects collected in northwest province of Esmeraldas were *T.dispar* L and many of them were collected in domestic areas. Studies related to vector biology and *T.cruzi* genotyping in vectors, human beings and wild reservoirs are required to understand the dynamics of *T.cruzi* transmission in this particular area since triatomine vectors had been shown an ease move from wild to domestic areas [9].
